# Computational discovery of *cis*-regulatory modules in *Drosophila *without prior knowledge of motifs

**DOI:** 10.1186/gb-2008-9-1-r22

**Published:** 2008-01-28

**Authors:** Andra Ivan, Marc S Halfon, Saurabh Sinha

**Affiliations:** 1 Department of Computer Science and Institute for Genomic Biology, University of Illinois at Urbana-Champaign, N. Goodwin Ave, Urbana, IL 61801, USA; 2 Department of Biochemistry, State University of New York at Buffalo, Main St, Buffalo, NY 14214, USA; 3 New York State Center of Excellence in Bioinformatics and the Life Sciences, Ellicott St, Buffalo, NY 14203, USA

## Abstract

We consider the problem of predicting *cis*-regulatory modules without knowledge of motifs. We formulate this problem in a pragmatic setting, and create over 30 new data sets, using *Drosophila *modules, to use as a 'benchmark'. We propose two new methods for the problem, and evaluate these, as well as two existing methods, on our benchmark. We find that the challenge of predicting *cis*-regulatory modules *ab initio*, without any input of relevant motifs, is a realizable goal.

## Abstract 2

Prediction of *cis*-regulatory modules *ab initio*, without any input of relevant motifs, is achieved with two novel methods.

## Background

Understanding the richness and complexity of the transcriptional network underlying the early stages of fruitfly development is a success story of developmental molecular biology. It is also an inspiration for bioinformaticians working on sequence analysis. This transcriptional regulatory network is implemented through '*cis*-regulatory modules' (CRMs), which are approximately 500-1,000 bp long sequences in the vicinity of genes harboring one to many binding sites for multiple transcription factors. These CRMs serve to mediate the activating and repressing action of the different transcription factors, and enforce the complex expression pattern of the adjacent gene. Discovery and analysis of CRMs is, therefore, a crucial step in understanding gene regulatory networks in the fruitfly and, more generally, in metazoans.

Starting with early advances [1-3], a host of computational approaches to discover CRMs in a genome have been proposed recently [4-8]. These methods typically rely on prior characterization of the binding affinities ('motifs') of the relevant transcription factors. For instance, one may search for CRMs involved in anterior-posterior segmentation of the embryo, if one knows the five to ten key transcription factors orchestrating this process, as well as their binding site motifs. However, the more common scenario, arising whenever one explores a relatively uncharted regulatory network, is that the relevant transcription factors and their motifs are unknown. The usual strategy of looking for clusters of (putative) binding sites is inapplicable, because we do not have a way to predict the binding sites in the first place. We explore here this more common version of the CRM prediction problem, where the relevant motifs are unknown.

Clearly, the new problem is less tractable than its traditional version with known motifs, and the 'genome-wide scan' approach of programs like Cis-analyst [1], Ahab [6], Stubb [7], or Cluster-Buster [4] seems infeasible. We therefore investigate a special variant of the problem, where the entire genome is not scanned; rather, the regions around a small set of genes are searched. To define this problem variant, we need to understand the notion of a 'gene battery'. This term was used by Britten and Davidson [9] to refer to a group of genes that are coordinately expressed because their regulatory regions respond to the same transcription factor inputs (also see [10].) In molecular terms, a gene battery is a group of genes that are regulated by CRMs containing similar transcription factor binding sites. The CRMs associated with genes in a battery are usually not identical in terms of either number or arrangement of binding sites, nor do they harbor sites for exactly the same set of transcription factors. Nevertheless, these CRMs share some level of similarity in terms of the collection of binding sites present within, and this similarity may be the basis for their computational discovery *ab initio*. This gives us the crucial insight to attempt CRM prediction in the absence of motifs. The gene battery CRM discovery problem is defined as: given a gene battery, and the 'control regions' of each gene, find in these control regions the CRMs that coordinate the expression of genes in the battery.

Here, the control region of a gene is the candidate sequence in which we must search for a gene's CRMs. A possible definition of a gene's control region may be 'the 10 Kbp sequence upstream of the gene', since CRMs are often found to be located in these regions. A more inclusive definition might be 'the 10 Kbp upstream and downstream sequences, and introns'. Under the new definition of the CRM discovery problem, we do not search the entire genome with known motifs; instead, we harness our prior knowledge about gene co-expression to narrow down the search space to the control regions of a gene battery.

It is clear that the gene battery CRM discovery problem is a highly practical problem with immense applicability in genomic biology. It is very common that a biologist has microarray data providing information on co-expressed clusters of genes. Such gene sets may be treated as a gene battery, and the scientist may wish to find out how they are regulated. This is a classic example of the gene battery CRM discovery problem. Whole-mount *in situ *hybridization data [11] comprise another source for defining potential gene batteries. For instance, a biologist interested in *Drosophila *dorsal-ventral axis specification may take a set of genes whose *in situ *images show dorsal-ventral expression patterns in the embryo, treat these genes as a gene battery, and proceed to identify the CRMs that regulate the gene battery. Once the CRMs have been identified, more detailed analysis of the modules may be conducted through binding site analysis and computational motif discovery, or direct experimental tests of the expression pattern driven by them, for example, through reporter gene assays [12].

### Outline

This paper is a comprehensive investigation into the gene battery CRM discovery problem. We ask several questions related to this problem, assuming that the relevant motifs are unknown. What are the data sets available for testing solutions to this problem? How do we evaluate the performance of any given algorithm on a given data set? What are the existing computational methods to solve the problem? Can we design new algorithms to solve this problem? How do the existing and new algorithms perform on the data sets?

In a previous study [13], we explored CRM properties and found that CRMs belonging to different gene batteries can have distinct characteristics. Our data indicated that several existing approaches to computational CRM discovery would be effective only for finding CRMs of certain subtypes, suggesting that CRM discovery methods need to be evaluated on a diverse selection of data sets. We show here how to use the REDfly database [14] to construct useful data sets for this purpose and present a 'benchmark' collection of 33 such data sets, marking a great leap (of coverage) from the currently available 2-3 data sets. We define normalized measures to evaluate the performance of any CRM prediction method. We identify and evaluate existing approaches for the problem, such as the 'CisModule' program of Zhou and Wong [15], and the Markov chain-based approach of Grad *et al*. [16]. We then propose and assess two novel algorithms for the problem, based on statistical properties of CRMs that we have reported in previous work [13,17]. The hallmark of each of these algorithms is that CRM prediction does not depend on accurate motif discovery, which is a notoriously difficult problem [18]. This marks a clear departure from previous methods like CisModule and EMCModule [19], where motif-finding and CRM discovery are tightly coupled. We find that our two new methods achieve significant accuracy on a majority of the benchmark data sets, despite not using any input motifs. This gives us the first clear indications that *ab initio *CRM prediction may be a realizable goal in several gene batteries, beyond the two or three widely studied examples (*Drosophila *segmentation [12] and human muscle-specific [20] or liver-specific [21] enhancers), where motifs were either known *a priori *or relatively easy to discover.

Our work opens up a new line of research by clearly focusing on a practical version of the CRM discovery problem, creating extensive benchmarks for it, and providing effective strategies and novel insights for attacking the problem.

### Related work

The literature on computational CRM discovery is dominated by algorithms that require well-characterized motifs [1-8,22,23]. One such example is our previously published algorithm, called 'Stubb' [7], which uses a probabilistic model parameterized by the given motifs to predict CRMs in a genome-wide scan. However, there are very few prior studies on the problem in the absence of motif information. Not surprisingly, each of these studies, discussed below, is designed for the 'gene battery CRM discovery problem', rather than genome-wide search.

To our knowledge, one of the first attempts to solve the gene battery CRM discovery problem was made by Grad *et al*. [16]. Their 'PFRSearcher' program used Gibbs sampling to find CRMs in control regions of *Drosophila *segmentation genes. However, no other gene batteries were tested in that work, making it unclear if the approach is generalizable. (Our previous work [13] found that this gene battery has CRMs with unique sequence characteristics that may not be representative of CRMs in other gene batteries.) Also, the PFRSearcher method relied crucially on inter-species comparison. Another algorithm to leverage evolutionary comparisons for CRM prediction (without motif knowledge) is called 'CisPlusFinder', developed by Pierstoff *et al*. [24]. More recently, Sosinsky *et al*. [25] have proposed a method that uses pattern discovery from seven *Drosophila *genomes to predict CRMs genome-wide, followed by validation on a data set of blastoderm segmentation-related CRMs. The method development and assessment in our work is exclusively based on a single genome. We recognize the potential of evolutionary information for CRM discovery, but this being a complex, phylogeny-dependent issue, we leave it for future research.

A model-based approach to CRM discovery (without motif knowledge) has been espoused by Zhou and Wong [15], whose CisModule program learns the motifs and the CRMs simultaneously from the data. The underlying idea is that spatial clustering of binding sites in a CRM should aid motif discovery, and that motif discovery should aid CRM prediction. Hence, both steps are performed in a combined probabilistic framework. The EMCModule program of Gupta and Liu [19] is similar; however, it begins with a generously large set of motifs (from a motif database or a separate motif-finding program), and learns which ones are relevant to the gene battery, and where the CRMs are located. Both these methods (CisModule and EMCModule) intertwine the motif discovery and CRM discovery tasks together. These programs have been shown to discover functional motifs and binding sites related to *Drosophila *segmentation, but were not tested for discovery of entire (experimentally delineated) CRMs. Also, the tests were performed on the two to three popular data sets available then and, hence, did not provide a comprehensive evaluation. The Gibbs Module Finder program of Thompson *et al*. [26] is another model-based approach in this genre. However, this work uses the term '*cis*-regulatory module' in a different manner, that is, to mean any region with at least two binding sites with a spacing of less than 100 bp. This definition is rather distinct from our semantics of a CRM, which is based on the expression pattern driven by the CRM rather than its binding site architecture. The Gibbs Module Finder was tested on a single gene battery (human skeletal muscle genes), and shown to find known binding sites and pairs thereof. This does not automatically imply its applicability to our problem setting.

There is another variant of the CRM discovery problem, which we do not address here. This is the 'supervised learning' approach of Chan and Kibler [27] or Nazina and Papatsenko [28] (also explored by Grad *et al*. [16]), where a set of known CRMs is available as 'training data'. These programs use such known CRMs to train their parameters before predicting new CRMs in any test sequences.

In summary, the gene battery CRM prediction problem is a relatively less studied, yet highly practical formulation of computational CRM discovery. There exist only a handful of methods, outlined above, that may be applied to this problem, but no such method has been tested on a large collection of data sets. The model-based approaches that have been proposed previously have focused on prediction of binding sites (and motifs), and have used the notion of CRMs as an aid to this discovery process. Here, our objective is to predict the CRMs themselves rather than their constituent binding sites or motifs.

## Results

### Benchmarks for the gene battery CRM discovery problem

We first describe a classic example of this problem. In *Drosophila*, meticulous experimentation has led to a rich collection of CRMs involved in the gene battery for anterior-posterior segmentation of the blastoderm stage embryo [29,30]. We refer to this set of approximately 50 CRMs as the blastoderm set of CRMs. All CRMs in this set drive some pattern of gene expression along the anterior-posterior axis, at the blastoderm stage of development. Their target genes, and respective control regions, make for a natural data set to evaluate CRM prediction methods. Indeed, the blastoderm set has been extensively used as a 'benchmark' in the past [1,2,6,7]. Here, our goal was to create several new benchmarks similar to this classic example.

The REDfly database [14] is an up-to-date, comprehensive collection of experimentally verified CRMs in *Drosophila *mediating regulation in a broad spectrum of gene batteries. The database also records the gene expression pattern driven by each CRM. We grouped REDfly CRMs based on common gene expression annotation, and took their target genes to be a gene battery. The natural way to construct a data set is to take the control regions of each of these genes. However, this choice makes the task of evaluating CRM predictions complicated, for the following reasons.

It has been widely observed, especially in the context of the blastoderm set of modules, that a control region may have multiple CRMs. In general, some of these may be unknown. Therefore, we will not know for sure if predictions that do not coincide with the known CRMs are true or false positives.

If multiple known CRMs lie in the same control region, the prediction task is more demanding than when each control region has exactly one CRM. The predictor has to have the additional ability to decide if there are one or more CRMs in any particular input sequence. In our first take on the problem, we wish to circumvent including this ability in our assessment, in order to simplify the evaluation.

Using the native control regions of the gene battery allows us less control on the 'difficulty level' of a data set. Some control regions will have a substantially greater ratio of signal (CRM positions) to noise (non-CRM position) compared to other sequences. While this is indeed a fact of real genomes, in this initial evaluation we want to have data sets where every input sequence has the same 'signal-to-noise' ratio.

We address the above issues in our design of data sets. Once the set of CRMs (with common expression annotation) have been decided, we plant each CRM in a carefully chosen artificial 'control region', built from the genome itself. This control region is constructed from the non-coding part of the *D. melanogaster *genome, and is required to have G/C content similar to the native context of the CRM. By constructing data sets in this manner, we minimize the chances of uncharacterized CRMs influencing the false positive estimation. The non-native control region still has the odd chance of containing an uncharacterized CRM, but it is extremely unlikely that such a CRM will be in the same gene battery as the planted CRMs of the data set. We create one control region for each CRM, requiring each control region (with CRM planted within) to be of a length ten times the length of the CRM. These choices were dictated by our need to 'standardize' the difficulty of the benchmark data sets, as discussed above. Given that a typical CRM has a length of approximately 500-1,000 bp, and a typical control region is 5-10 Kbp long, a 1:10 ratio of CRM length to total length seems realistic.

We obtained 33 data sets in this manner, with 4-77 sequences (an average of 16) in a data set, and where the CRM lengths range from 83 bp to 2,013 bp. Details of these data sets are presented in Table 1. The entire collection of data sets is available in Additional data file 1. Note that each data set name is prefixed by a 'mapping number', which we explain now. Data sets were constructed using the expression pattern information provided in REDfly, by grouping CRMs with similar tissue specificity. Different mappings represent different levels of tissue specificity, and correspond to Figures S1-1b, S1-1c, and S1-2 in Li *et al*. [13]. 'Mapping3' represents the highest level clustering of CRMs, such as 'adult' or 'larva'. On the other hand, 'mapping1', represents the lowest level of tissue specificity, such as 'ventral ectoderm' or 'cardiac mesoderm'. 'Mapping2' is an intermediate level of specificity. Thus, for example, 'mapping2.mesoderm' includes all CRMs that regulate gene expression in the mesoderm, whereas in mapping1 these CRMs are divided between 'adult mesoderm', 'cardiac mesoderm', 'larval mesoderm', 'somatic mesoderm' and 'visceral mesoderm'. Mappings at different levels may refer to the same tissue (for example, mapping1.mesoderm and mapping2.mesoderm), in which case the mapping with the higher numbering refers to a more inclusive definition of specificity to that tissue. We also note that data sets defined by us are potentially non-exclusive, that is, the same CRM can belong to more than one data set. This is possible if the CRM regulates expression in more than one tissue, or if one data set is subsumed by another data set at a higher level mapping.

**Table 1:** Statistics for the data sets in our benchmark

Name	Number of CRMs	Minimum CRM length	Maximum CRM length	Average CRM length	Total CRM length (Kbp)
mapping3.adult	34	83	2,013	748	25
mapping1.adult mesoderm	5	126	927	561	2
mapping1.amnioserosa	7	469	1,500	708	4
mapping1.blastoderm	77	126	1,833	906	69
mapping1.cardiac mesoderm	8	237	1,513	536	4
mapping1.cns	34	304	1,986	1,034	35
mapping1.dorsal ectoderm	8	267	1,657	842	6
mapping1.ectoderm	37	105	2,015	839	31
mapping2.ectoderm	51	105	2,015	815	41
mapping1.endoderm	16	220	1,373	579	9
mapping1.eye	6	187	1,930	824	4
mapping2.eye	18	187	2,015	868	15
mapping1.fat body	5	375	529	456	2
mapping1.female gonad	10	83	1,657	442	4
mapping1.glia	7	515	1,890	899	6
mapping1.imaginal disc	47	177	2,015	938	44
mapping2.imaginal disc	12	490	2,015	1,248	14
mapping3.larva	69	176	2,015	892	61
mapping1.male gonad	8	200	1,319	862	6
mapping1.malpighian tubules	4	540	1,373	782	3
mapping1.mesectoderm	5	601	1,415	913	4
mapping1.mesoderm	16	105	1,415	544	8
mapping2.mesoderm	45	105	1,513	518	23
mapping1.neuroectoderm	7	343	1,360	575	4
mapping2.neuronal	54	177	2,013	988	53
mapping1.pns	24	177	2,013	976	23
mapping2.reproductive system	21	83	1,801	734	15
mapping1.salivary gland	6	295	1,890	786	4
mapping1.somatic muscle	12	312	1,513	718	8
mapping1.tracheal system	9	515	2,015	1,236	11
mapping1.ventral ectoderm	12	343	1,657	700	8
mapping1.visceral mesoderm	12	183	1,104	451	5
mapping2.wing	33	177	2,015	1,029	33

Each control region is ten times the CRM length.

### Performance evaluation

Each data set consists of a set of control regions, with a single CRM located within each control region. In evaluating any module prediction algorithm, we require it to predict one CRM per input sequence, and that each predicted module be of the same length (for reasons explained below). This length, calculated as the mean of the known CRM lengths in the data set, is given as input to the prediction tool. Most tools evaluated here conform to these requirements, with the exception of CisModule. This program can predict multiple, variable-length CRMs per sequence, and its output is post-processed (as described in Materials and methods) to meet our requirements.

For each data set, we have a set of positions (*I*_*k*_) known to be CRM positions, and a set of positions (*I*_*p*_) predicted by a method. We may compute the positive predictive value ppv (or precision) and sensitivity sens (or recall) as per the following formulas:

(1) $$ppv=\frac{\left|I_{k}\cap I_{p}\right|}{\left|I_{p}\right|}sens=\frac{\left|I_{k}\cap I_{p}\right|}{\left|I_{k}\right|}$$

Note that by design of the experiments, we have |*I*_*p*_| = |*I*_*k*_|, making the precision and recall identical. This convenient scenario was the motivation behind choosing the mean CRM length as the window length input to the evaluated methods. It lets us avoid having to compare different methods that may outdo each other on one of these dimensions (precision or recall). In real-world applications, a program has to predict not only the locations of CRMs but also their lengths. However, here we chose not to test the ability to predict CRM lengths, by requiring each program to predict CRMs of a given length. This desired CRM length was made equal for all control regions, to mimic real applications where the true CRM lengths are not known *a priori*.

In light of the above discussion, the sensitivity sens is used as the measure for performance in the rest of this paper. The sensitivity allows us to compare the performance of several methods on the same data set, but is not comparable across data sets. The expected sensitivity of a random prediction depends on several aspects of the data set, most notably its total length. Therefore, to normalize against this chance expectation, we compute an 'empirical *p*-value' of the sensitivity, as follows. We randomly select in each control region a window of the same length as the module prediction. The sensitivity of this random set of window locations is calculated, the process is repeated 100,000 times, and the empirical *p*-value is defined as the fraction of times that the sensitivity was greater than that observed for the actual predictions. We consider the predictions of any method to be significant if its sensitivity *p*-value is less than 0.05.

#### Maximum sensitivity

We note that due to the way the evaluation is done, and because of the variable lengths of the true CRMs, a sensitivity of 100% is usually impossible to achieve. If the predicted CRM lengths are always of length equal to the mean CRM length, the modules longer than this mean length cannot be predicted entirely. Therefore, when reporting results on a data set, we also note the maximum sensitivity achievable on that data set. We point out that the sensitivity *p*-value automatically accounts for the fact that a 100% sensitivity is usually not achievable.

#### CRM-level sensitivity

Apart from the nucleotide-level sensitivity, we also assess sensitivity at the CRM level, as follows. We declare a predicted module (in a control region) as a 'hit' if its overlap with the known module is at least half as long as the smaller of the two known and predicted modules. We then count the number (and percentage) of hits in a data set, and call it the 'CRM-level sensitivity'. This measure has an intuitive appeal, since partial identification of the module is often enough for follow-up experiments to refine upon. Also, some of the known CRMs are likely to be 'too long', that is, the true CRM is only a part of the annotated delineation [13]. In such cases, even perfectly accurate predictions would earn less than 100% sensitivity at the nucleotide level. Considering the CRM-level sensitivity addresses this issue.

### Existing methods and their performance

#### Stubb

We begin our evaluations with a program that uses the knowledge of motifs to scan for modules, since this is currently the standard approach to CRM discovery, and provides a useful reference point for programs that do not rely on known motifs. The Stubb program [7] takes a set of known position weight matrix (PWM) motifs and scans the input sequences in sliding windows of a fixed length. It scores each such window by its likelihood of being generated by a certain probabilistic model parameterized by the input PWMs. In our tests, the highest scoring window in each control region was considered as Stubb's prediction. As a preliminary test, we evaluated Stubb on the well-studied blastoderm data set (mapping1.blastoderm) of 77 CRMs, using a small set of 8 PWMs known to regulate this gene battery. We obtained a sensitivity of 46% (compared to a maximum achievable sensitivity of 77%), with *p*-value ~0. This is consistent with the expectation that knowledge of relevant motifs leads to high accuracy. We also point out that a sensitivity of 46%, though not phenomenal in its absolute value, is highly significant, and represents the state-of-the-art in motif-driven CRM prediction. Such predictions have been reported in the literature to lead to novel CRM discoveries [12].

For the remaining data sets of our benchmark, we typically do not know the relevant motifs. Hence, in the full-scale evaluation on all data sets, Stubb was run with a large collection of 53 PWMs from the FlyREG database (see Additional data file 1 for a list of these PWMs). Most of these 53 motifs will be largely irrelevant to any particular data set, and may cause Stubb to predict biologically incoherent combinations of transcription factor binding sites as modules. The sensitivity of Stubb predictions and their empirical *p*-values are shown in Table 2. Stubb performed significantly well (*p*-value ≤0.05) on 12 of the 33 data sets. These results, from an approach where the relevant motifs are not known, but a modest collection of motifs is utilized, provide an interesting base line for other approaches, where no motif information is utilized.

**Table 2:** Performance of Stubb, D2Z-set, and CSam on 33 data sets in our benchmark

				Stubb^§^		D2Z-set^§^		CSam^§^	
						
Data set	Sequence number*	Length^†^	Maximum sensitivity^‡^	*P*-value	Sensitivity	*P*-value	Sensitivity	*P*-value	Sensitivity
MAPPING3.ADULT	34	254,800	0.71	**0.01**	0.20	0.72	0.07	0.15	0.13
mapping1.adult mesoderm	5	28,085	0.76	0.51	0.05	0.11	0.22	0.51	0.05
mapping1.amnioserosa	7	49,635	0.84	0.25	0.15	0.34	0.12	0.09	0.23
MAPPING1.BLASTODERM	77	698,840	0.77	**0.00**	0.36	0.10	0.13	**0.00**	0.26
MAPPING1.CARDIAC MESODERM	8	42,979	0.76	0.08	0.22	**0.03**	0.28	0.12	0.19
MAPPING1.CNS	34	352,108	0.80	0.48	0.10	**0.01**	0.20	**0.02**	0.18
mapping1.dorsal ectoderm	8	67,490	0.77	0.08	0.22	0.88	0.00	0.08	0.22
MAPPING1.ECTODERM	37	311,000	0.72	**0.01**	0.20	**0.00**	0.20	**0.00**	0.21
MAPPING2.ECTODERM	51	416,473	0.74	**0.01**	0.18	**0.05**	0.15	**0.00**	0.23
MAPPING1.ENDODERM	16	92,723	0.82	**0.01**	0.24	0.31	0.12	**0.01**	0.26
MAPPING1.EYE	6	49,494	0.70	1.00	0.00	0.48	0.08	**0.02**	0.32
mapping2.eye	18	156,531	0.69	0.19	0.14	0.68	0.07	0.88	0.04
mapping1.fat body	5	22,831	0.93	0.14	0.20	1.00	0.00	0.45	0.09
MAPPING1.FEMALE GONAD	10	44,269	0.62	**0.03**	0.24	0.97	0.00	0.86	0.02
mapping1.glia	7	63,008	0.82	0.49	0.09	0.16	0.19	0.21	0.17
MAPPING1.IMAGINAL DISC	47	441,597	0.77	0.55	0.09	**0.00**	0.20	0.24	0.12
mapping2.imaginal disc	12	149,915	0.80	0.57	0.08	0.12	0.18	0.33	0.12
MAPPING3.LARVA	69	616,635	0.76	**0.05**	0.14	**0.02**	0.15	**0.00**	0.18
mapping1.male gonad	8	69,044	0.85	0.22	0.15	0.46	0.10	0.15	0.18
mapping1.malpighian tubules	4	31,338	0.81	0.10	0.25	1.00	0.00	0.30	0.16
MAPPING1.MESECTODERM	5	45,712	0.83	0.18	0.20	0.43	0.10	**0.00**	0.46
MAPPING1.MESODERM	16	87,140	0.72	**0.02**	0.21	0.09	0.17	0.22	0.13
MAPPING2.MESODERM	45	233,441	0.75	**0.00**	0.22	**0.00**	0.20	**0.02**	0.16
MAPPING1.NEUROECTODERM	7	40,315	0.80	**0.01**	0.34	1.00	0.00	**0.00**	0.51
MAPPING2.NEURONAL	54	534,081	0.78	0.24	0.12	**0.00**	0.19	**0.00**	0.26
MAPPING1.PNS	24	234,532	0.78	**0.03**	0.19	0.07	0.17	**0.01**	0.21
mapping2.reproductive system	21	154,400	0.69	0.16	0.14	0.34	0.10	0.24	0.12
mapping1.salivary gland	6	47,232	0.74	0.55	0.06	1.00	0.00	0.36	0.11
MAPPING1.SOMATIC MUSCLE	12	86,317	0.79	0.29	0.12	0.05	0.21	**0.01**	0.28
mapping1.tracheal system	9	111,351	0.85	0.55	0.08	0.21	0.16	0.18	0.17
MAPPING1.VENTRAL ECTODERM	12	84,154	0.77	**0.00**	0.38	0.32	0.12	**0.01**	0.27
MAPPING1.VISCERAL MESODERM	12	54,278	0.77	0.46	0.10	0.32	0.12	**0.01**	0.28
MAPPING2.WING	33	340,094	0.78	0.14	0.13	**0.00**	0.23	**0.00**	0.22

*The number of sequences in a data set; ^†^the total sequence length; ^‡^the maximum sensitivity possible. ^§^The sensitivity and its empirical *p*-value are given for each method tested. Data set names are capitalized if at least one of the three methods performs significantly (*p*-value ≤0.05; shown in bold) on it.

The program EMCModule [19] has functionality that is similar to Stubb, and uses a given database of motifs to find CRMs. Due to its similarities with Stubb, we chose not to evaluate this program here, instead focusing on Stubb, a program we are much more familiar with.

#### CisModule

CisModule is a powerful CRM prediction program that does not require input motifs: it attempts to learn the relevant PWMs while searching for modules. When run on our benchmark with default settings, we found CisModule to consistently overpredict modules, leading to very low positive predictive value (PPV; precision) and very high sensitivity (data not shown). Since our evaluations require every method to predict a single, fixed-length window in each control region, we then processed CisModule's output as described in Materials and methods. The result, however, was that the prediction was significant (sensitivity *p*-value ≤0.05) on only one data set. (Table S1 in Additional data file 1.) We explored alternative settings of the CisModule parameters (such as five motifs instead of three), but the results were similar.

The poor performance of CisModule on our data sets is possibly the result of an incorrect choice of parameters (we used default parameters), or our post-processing step that forces a fixed length window to be predicted in each input sequence, or both. More insight into the workings of this program should lead to better predictions, which we leave as a future exercise. It is also worth noting that CisModule has been tested [15] previously as a 'motif finding application' that uses clustering of binding sites to improve the extremely difficult motif finding task. In a separate paper [31], the authors used the CisModule-predicted motifs as input to another program called CisModScan, which searches for significant clusters of matches to the motifs, similar to Stubb. Our preliminary tests with this strategy, followed by the post-processing step to obtain equal length predicted CRMs, did not show improved performance. Again, we speculate that a carefully designed combination of CisModule and CisModScan may provide high performance accuracy in our data sets. The public availability of our benchmark and evaluation tools will greatly facilitate testing of CisModule and similar methods by other researchers.

#### Markov chain discrimination method

The 'Markov chain discrimination' (MCD) method is our implementation of the 'PFRSampler' algorithm of Grad *et al*. [16]. This method considers the word frequency distribution in the given set of candidate CRMs and a set of background sequences, and uses a Markov chain approach to discriminate between the two. More specifically, the MCD score is obtained by training a fifth order Markov chain on the given set of sequences, evaluating the likelihood of these sequences being generated by the trained Markov chain, and contrasting this likelihood to the likelihood of their generation by a null (background) model. The stronger the contrast, the more different the sequences are from the background, and the higher their chances of being CRMs. Our implementation uses a simulated annealing search strategy to find the highest scoring set of windows in the control regions. Details of the algorithm are presented in Materials and methods. We note that unlike the original PFRSampler algorithm, which exploits evolutionary conservation, our implementation is designed for single species data. The MCD method performed significantly well on only 3 of the 33 data sets, and its sensitivity *p*-values are shown in Table S1 in Additional data file 1.

### Design of new methods

We designed and implemented two new strategies for the gene battery CRM discovery problem that do not require given PWM motifs. In fact, their common theme is that they do not attempt to discover accurate PWMs as part of their module search. We briefly describe these new methods next. Details are presented in Materials and methods.

#### CSam

We propose a new strategy, called CSam (short for CRM Sampler; pronounced see-sam), to predict CRMs in given control regions. Here, a set of candidate CRMs is evaluated by the number of statistically overrepresented short words in that set. The intuition is that if a set of CRMs share binding sites for the same factor, this will cause many short words (that are similar to the true binding motif for the factor) to be statistically overrepresented. Note that all overrepresented words in a set of CRMs may not represent transcription factor binding motifs, nor are we interested in determining which words are real motifs; all that matters is that the count of such words be greater in a collection of related CRMs than in random windows of the same size. The new approach is motivated by our recent work [13], where we found the count of overrepresented words to be significantly higher in CRMs than in random non-coding sequences.

As a design principle in CSam, we avoid determining the precise form of the true motif(s), for example, learning a few distinct, high-confidence PWMs. (This 'motif-finding' problem has been demonstrated empirically to be extremely hard to solve [18].) We instead rely on broad statistical effects of the shared binding sites on the word frequency distribution in the set of CRMs. This is what sets this method clearly apart from the other approaches to this problem, such as CisModule or EMCModule. Also, there is no need in this approach to know the number of distinct functional motifs *a priori*. With a clearly defined score for any set of candidate CRMs, the CSam algorithm searches for the highest scoring set using a technique called 'simulated annealing' (see Materials and methods). We also experimented with a different search strategy, namely, 'Gibbs sampling' in conjunction with the same scoring scheme.

#### D2Z-set

In the D2Z-set method, we make use of our previous work [17] on measuring the similarity between any two regulatory sequences based on their word frequency distributions. In a set of functionally related CRMs (for example, those belonging to a gene battery), many or all pairs of CRMs should share binding sites. The challenge is to capture the resulting similarity between CRMs by a suitable statistical measure. The 'D2 score' [32] is the number of *k*-mer matches between two given sequences, and the 'D2Z score' introduced in our earlier work [17] computes the statistical significance (z-score) of this number. The z-score is a way to normalize the raw D2 score for dependence on the nucleotide frequencies ('background models') of the sequences. The D2Z score was found in [17] to perform favorably in comparison to a modest number of existing methods for alignment-free sequence comparison [33,34].

The D2Z score measures the similarity between two sequences that results from the shared binding sites within them. Here, we build upon this pairwise measure to develop a score for an arbitrary set of candidate CRMs, called the 'D2Z-set' score (see Materials and methods). We then devised a search algorithm based on 'simulated annealing' that looks for the highest scoring set in the given control regions. This entire method is called the 'D2Z-set' method.

### Performance of new methods

The sensitivity *p*-values for CSam and D2Z-set, along with those of Stubb, are shown in Table 2. At a *p*-value threshold of 0.05, we expected each method to perform significantly well on two sets on average. CSam performs significantly on 16 of the 33 data sets, while D2Z-set does so for 9 data sets. Both compare well with Stubb's predictions (significant for 12 data sets). Of particular interest is the observation that CSam outperforms Stubb in these tests. This suggests that if the set of PWMs relevant to a gene battery are not known, it may be more advantageous to predict CRMs using a motif-agnostic method (CSam), as compared to a state-of-the-art motif-driven approach (Stubb) that relies on a broad collection of PWMs.

We first make a few observations on Table 2. Firstly, we consider the performance figures for the new motif-agnostic methods CSam and D2Z-set, and find as many as 25 (of the 33 × 2 = 66 entries) to be 0.05 or below. To get a rough idea of how significant this is, consider these numbers as independently obtained *p*-values (which should follow a uniform distribution): one would expect 0.05 × 66 = 3 entries at 0.05 or below. Secondly, we note to what extent the different methods perform well on the same data sets. This is shown in Table 3. We find a substantial overlap (Hypergeometric test, *p *< 0.03) among the data sets on which CSam and D2Z-set perform well. In fact, there is only one data set on which D2Z-set performs significantly and CSam does not. Similarly, there is a significant overlap (Hypergeometric test, *p *< 0.06) between the data sets on which Stubb and CSam perform well.

**Table 3:** Entry for any pair of methods is the number of data sets on which both methods performed significantly well (sensitivity *p*-value <0.05)

	Stubb	CSam	D2Z-set
Stubb	12	9	4
CSam	-	16	8
D2Z	-	-	9

Diagonals indicate the number of data sets on which the corresponding method performed well.

We also noted, from Table 2, that data sets with larger numbers of CRMs tended to show better performance overall. To quantify this, we partitioned the 33 data sets into those where at least one of the two methods (CSam or D2Z-set) performed significantly well, and those where neither method performed well. The data sets in the second partition were significantly smaller than those in the first (Wilcoxon rank-sum test, *p *< 0.009).

Next, we turn our attention to the raw values of the sensitivities achieved on these data sets. Limiting ourselves to the cases where the *p*-value is significant, we find that CSam achieves a raw sensitivity in the range 16-51%, at an average of 27%. Recall that due to the way our tests are designed, a 100% sensitivity is often impossible to achieve; in fact, as Table 2 reveals, the maximum possible sensitivity is about 77% on average. Next, to get an idea of the practical importance of the observed sensitivity levels, consider a typical 500 bp module in a typical 5,000 bp control region. A sensitivity of approximately 27% means that the predicted window overlaps the known module in about 135 positions. To be able to find the location of the module to this resolution, in a 5,000 bp search region, is clearly useful from a biological perspective. The precise delineation of that module may be recovered from follow-up experiments.

**Figure 1:**
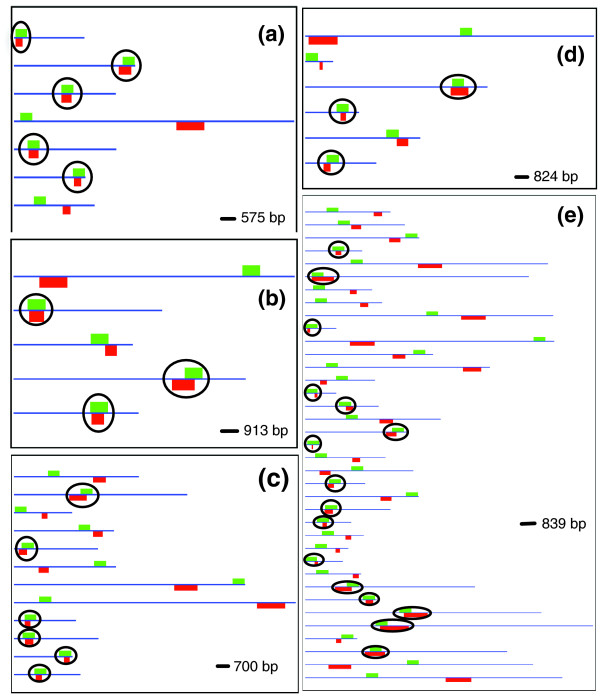
Performance of CSam on five data sets where its sensitivity *p*-value was below 0.05. The data sets are **(a) **mapping1.neuroectoderm, **(b) **mapping1.mesectoderm, **(c) **mapping1.ventral ectoderm, **(d) **mapping1.eye and **(e) **mapping1.ectoderm. In each panel, every sequence is shown as a blue line, the location of a known module is shown as a red rectangle below the line and the location of a predicted module is shown as a green rectangle above the line. The displays of different panels are to different scales.

We next look at the performance of our CRM prediction methods pictorially, to get a better understanding of the sensitivity values of Table 2. Figure 1 shows the known and CSam-predicted modules in five different data sets. These are selected from the data sets where CSam performed significantly well (*p *< 0.05), but with raw sensitivity values ranging from 0.21 to 0.51. The plotted data sets are a representative sample, and not the ones with the five highest sensitivity values. Figure 1a ('mapping1.neuroectoderm') has the highest sensitivity (0.51), and we see that the known CRM (red rectangle below line) is correctly predicted (green rectangle above line) in five of the seven sequences (these cases are marked with ovals). Note that even though the nucleotide level sensitivity is 51%, the method has identified 71% of the modules in the data set. We find the same theme in the other data sets shown in Figure 1. Thus, the mapping1.mesectoderm data set (Figure 1b) has three of five (that is, 60%) of its modules correctly identified while the nucleotide-level sensitivity is 46%. The next two panels (Figure 1c,d) show mapping1.ventral_ectoderm and mapping1.eye, where CSam has sensitivity values of 27% and 32%, respectively. In these two data sets, the percentage of modules discovered is 50% (6 of 12, and 3 of 6, respectively). Finally, we look at the data set mapping1.ectoderm (Figure 1e), which has 'only' 21% sensitivity, but at the CRM-level this translates to 16 of the 37 modules (that is, 43%) being correctly identified. Thus, visual inspection reveals that the data sets assessed as showing 'significant' performance indeed show a high rate of correct module discovery.

We next extended the above analysis to all data sets and methods. We counted the number (and percentage) of CRMs that are correctly predicted (as described in the section 'Performance evaluation'), thereby obtaining a CRM-level sensitivity. These results are shown in Table 4. We find CSam to provide the best CRM-level sensitivity for 18 of the 33 data sets - more than any other method, including the motif-driven program Stubb. Restricting ourselves to the 16 data sets in which CSam performed significantly well (sensitivity *p*-value <0.05), we find 13 data sets (81%) to have a CRM-level sensitivity of 30% or above, and 6 data sets (38%) to have over 40% of their CRMs correctly predicted. This clearly shows that the statistically significant nucleotide-level sensitivity values of Table 2 correspond to high accuracy in predicting CRMs.

**Table 4:** CRM-level sensitivity of data sets

Set name	CRMs*	Stubb^†^	CSam^†^	D2Z-set^†^	CisModule^†^	MCD^†^
mapping3.adult	34	**0.35 **(12)	0.24 (8)	0.21 (7)	0.24 (8)	0.26 (9)
mapping1.adult mesoderm	5	0.20 (1)	0.20 (1)	**0.40 **(2)	0.00 (0)	0.20 (1)
mapping1.amnioserosa	7	0.14 (1)	**0.29 **(2)	0.14 (1)	0.00 (0)	0.29 (2)
mapping1.blastoderm	77	**0.53 **(41)	0.42 (32)	0.21 (16)	0.14 (11)	0.12 (9)
mapping1.cardiac mesoderm	8	0.38 (3)	0.25 (2)	**0.50 **(4)	0.12 (1)	**0.50 **(4)
mapping1.cns	34	0.12 (4)	**0.26 **(9)	0.24 (8)	0.15 (5)	0.15 (5)
mapping1.dorsal ectoderm	8	**0.38 **(3)	0.25 (2)	0.00 (0)	0.12 (1)	0.25 (2)
mapping1.ectoderm	37	0.30 (11)	**0.38 **(14)	0.35 (13)	0.22 (8)	0.19 (7)
mapping2.ectoderm	51	0.24 (12)	**0.39 **(20)	0.25 (13)	0.14 (7)	0.18 (9)
mapping1.endoderm	16	0.25 (4)	**0.31 **(5)	0.19 (3)	0.25 (4)	0.12 (2)
mapping1.eye	6	0.00 (0)	**0.50 **(3)	0.17 (1)	0.33 (2)	0.17 (1)
mapping2.eye	18	**0.33 **(6)	0.11 (2)	0.11 (2)	0.22 (4)	0.11 (2)
mapping1.fat body	5	**0.20 **(1)	0.00 (0)	0.00 (0)	0.00 (0)	0.00 (0)
mapping1.female gonad	10	**0.30 **(3)	0.10 (1)	0.00 (0)	0.20 (2)	0.00 (0)
mapping1.glia	7	0.14 (1)	0.29 (2)	0.29 (2)	0.14 (1)	**0.43 **(3)
mapping1.imaginal disc	47	0.11 (5)	0.21 (10)	**0.30 **(14)	0.19 (9)	0.15 (7)
mapping2.imaginal disc	12	0.08 (1)	0.17 (2)	0.17 (2)	0.08 (1)	**0.25 **(3)
mapping3.larva	69	0.16 (11)	**0.30 **(21)	0.25 (17)	0.19 (13)	0.13 (9)
mapping1.male gonad	8	**0.25 **(2)	**0.25 **(2)	0.12 (1)	0.12 (1)	0.12 (1)
mapping1.malpighian tubules	4	**0.25 **(1)	**0.25 **(1)	0.00 (0)	0.00 (0)	**0.25 **(1)
mapping1.mesectoderm	5	0.20 (1)	**0.60 **(3)	0.20 (1)	0.00 (0)	0.20 (1)
mapping1.mesoderm	16	**0.38 **(6)	0.31 (5)	0.31 (5)	0.31 (5)	0.31 (5)
mapping2.mesoderm	45	**0.36 **(16)	0.24 (11)	0.31 (14)	0.07 (3)	0.22 (10)
mapping1.neuroectoderm	7	0.43 (3)	**0.71 **(5)	0.00 (0)	0.29 (2)	0.14 (1)
mapping2.neuronal	54	0.15 (8)	**0.35 **(19)	0.24 (13)	0.09 (5)	0.09 (5)
mapping1.pns	24	0.25 (6)	**0.29 **(7)	**0.29 **(7)	0.17 (4)	**0.29 **(7)
mapping2.reproductive system	21	**0.29 **(6)	0.19 (4)	0.14 (3)	0.19 (4)	0.24 (5)
mapping1.salivary gland	6	**0.17 **(1)	**0.17 **(1)	0.00 (0)	**0.17 **(1)	0.00 (0)
mapping1.somatic muscle	12	0.25 (3)	**0.33 **(4)	**0.33 **(4)	0.17 (2)	0.08 (1)
mapping1.tracheal system	9	0.11 (1)	**0.22 **(2)	**0.22 **(2)	0.00 (0)	0.00 (0)
mapping1.ventral ectoderm	12	**0.50 **(6)	**0.50 **(6)	0.25 (3)	0.08 (1)	0.17 (2)
mapping1.visceral mesoderm	12	0.17 (2)	0.42 (5)	0.25 (3)	0.17 (2)	**0.50 **(6)
mapping2.wing	33	0.24 (8)	**0.30 **(10)	**0.30 **(10)	0.09 (3)	0.18 (6)

*The number of CRMs in a data set. ^†^The fraction (and number in parentheses) of CRMs in a data set that were 'hits' (that is, overlap greater than half the length of the shorter window). The best CRM-level sensitivity for each data set is in bold.

### Evaluation of scoring schemes

The two new methods CSam and D2Z-set, as well as the MCD algorithm, which is our implementation of an existing method, have two major components: the scoring scheme and the search strategy. We next sought to decouple these two components in our evaluations, and directly test the efficacy of the scoring scheme. The basic idea is to score the 'true set' of CRMs in a data set, and ask how high this score is when compared to the score of random sequence sets. More specifically, we compute the 'score *p*-value' for a given scoring scheme and a given data set, as follows. First, we score the set of CRMs in the data set, to obtain what we call the 'true solution score'. Second, we generate 100 random sets of sequences. Every random set contains the same number and length of sequences as the set of CRMs, the sequences being chosen at random from the non-coding genome. Finally, we score each of these random sets, and count what fraction of them is better than the true solution score. This is called the 'score *p*-value'. Clearly, a scoring scheme with a small 'score *p*-value' is one that effectively characterizes the CRMs of a gene battery.

The score *p*-value is a useful tool to evaluate new scoring schemes that may be devised in the future, even before they are coupled with a search algorithm into a complete CRM prediction program. For instance, it can help in quick evaluation of many different parameter settings of a new scoring scheme. The score *p*-values for each of the three scoring schemes (CSam, D2Z-set, and MCD) are presented in Table S2 in Additional data file 1. We observe that CSam, D2Z-set, and MCD have score *p*-values less than 0.05 on 12, 12 and 10 of the 33 data sets, respectively. In light of such comparable performance of the scoring schemes, and the search results from the previous section, it appears that the search strategy used by MCD has the most scope for improvement. Since the same search scheme (simulated annealing) is used by each of the three programs, we believe that this search scheme and the MCD scoring function are not ideally matched.

We also notice, in some cases, that the data sets on which the scoring scheme performs well (score *p*-value <0.05) are the data sets where the search was successful (sensitivity *p*-value <0.05). For the D2Z method, this association is statistically significant (Hypergeometric test, *p *= 0.011). It is also strong for the CSam method (*p *= 0.086), but weaker for the MCD method (*p *= 0.19). This echoes our observation above that the search strategy of MCD needs further improvement.

### Evaluation of search strategies

The 'score *p*-value' introduced above, after a slight modification, also allows us also evaluate the effectiveness of the search strategies. Recall that each of the new methods uses a sampling-based search scheme, which is not guaranteed to find the global optimum. Therefore, it is important to find out if the local optimum reported by each method (for each data set) is close to the global optimum. To do this, we treat the solution reported by a method as the 'true set of CRMs' and compute its score *p*-value as in the previous section. We find that for all three methods and all data sets, the empirically computed score *p*-value is 0. Thus, within the limits of our assessment, each method indeed finds the global optimum of its objective function, in each data set. Furthermore, the optimum in each data set is always higher scoring than the true solution. This, together with the findings of the previous section, makes the case for the design of better scoring functions, in order to improve CRM prediction accuracy.

### Effect of data set size

One expects that the CRM prediction methods would have more accurate predictions if the control regions were smaller, since the search space would be smaller. To investigate this, we designed data sets where the CRM size to total size was in the ratio of 1:5 instead of 1:10, and ran CSam (the top performing method) as before. The results are shown in Table S3 in Additional data file 1. We find the raw sensitivities to be higher in the new, smaller data sets for 28 of the 33 cases. This is expected at least partly, because a randomly placed window is expected to have greater sensitivity in the shorter control regions. However, the sensitivity *p*-value accounts for this change in random expectation, and we found that the count of sets with sensitivity *p*-values less than 0.05 grew from 16 to 20. This overall improvement in performance is because the search algorithm faces a less difficult task when given smaller input sets.

### Effect of homotypic clustering of binding sites

We considered the possibility that the CRM prediction methods rely heavily on repeated occurrences of the same short word within the same CRM, a phenomenon known as 'homotypic clustering of binding sites'. We adopted the methodology of our previous work [13] to show that successful CRM prediction on a data set is not reliant on homotypic clustering of sites in individual CRMs. We computed the 'FTTz' score [13] of each CRM (this is a measure of homotypic clustering), transformed it into a rank (percentile) with respect to the full complement of REDfly CRMs, and obtained the mean FTTz rank of CRMs in a data set. A data set with a high 'mean FTTz rank' comprises CRMs with a greater extent of homotypic clustering. We found the 33 data sets in our benchmark to have a mean FTTz rank of 50% (on average), and only one data set was above 70%. We then considered the data sets on which each method (Stubb, CSam, or D2Z-set) performed significantly well, and found these data sets to have a mean FTTz rank of 50.1% (Stubb), 52.3% (CSam) and 50.2% (D2Z-set), on average, clearly showing that the significant performance was not due to any unusual bias towards homotypic clustering in a data set.

### Effect of experimental design

In the tests above, CRM prediction was performed with the window length set to the mean CRM length for that data set. In a realistic scenario this number is unknown; hence, we repeated all tests with input window length set to the fixed value of 750 bp. The results, shown in Table S5 in Additional data file 1, reveal little change in performance (from those in Table 2), with Stubb, CSam and D2Z-set performing significantly on 12, 14, and 9 data sets, respectively. The identities of these data sets are almost unchanged from those in Table 2.

We also repeated our evaluations on data sets where CRMs were kept in their native genomic contexts, and control regions of a length ten times the CRM length were extracted around each CRM (Tables S6 and S7 in Additional data file 1). We expected the performance to degrade, potentially because of unknown CRMs present in the native control regions, and the fact that each program reports only one CRM per control region. Indeed, we find that Stubb, CSam and D2Z-set perform significantly well on 7, 8, and 4 data sets, respectively, which is about half as many as in our semi-real benchmark. At least one of these three methods did well on 14 of the 33 data sets. In a somewhat opposite trend, the MCD program shows significant performance on five data sets in this new setting, compared to three data sets in the original benchmark.

### Motif discovery improves after CRM prediction

**Figure 2:**
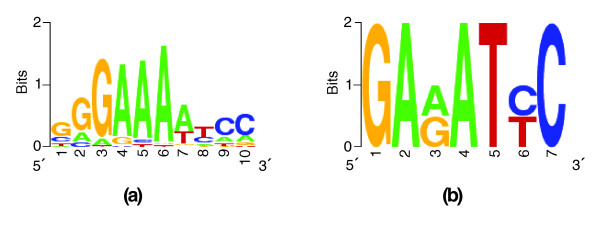
Logos of the known and predicted motifs in a data set. **(a,b)** The known *Dorsal* motif (a) and the motif discovered using YMF on *ab initio *predicted CRMs in the mapping1.neuroectoderm data set (b). The same motif finding program when run on the entire data set mapping1.neuroectoderm (which include the CRMs) did not find this or any other known motif from the FlyREG database.

*Ab initio *discovery of CRMs can be a vital step towards accurate motif-finding and prediction of transcription factor binding sites. This is because identification of the CRMs confines the motif search to a much smaller region of DNA than the input control regions. To illustrate this, we took the neuroectoderm data set, which shows the best CRM prediction as per the CSam method (sensitivity 0.51, *p*-value 0.00). We ran the YMF motif finding program [35] on the entire data set and the CRMs predicted by CSam for this data set. In each run, YMF was made to report the most significant motif of lengths 6, 7 and 8, respectively. The three motifs thus discovered computationally were then compared to a database of 53 known *Drosophila *motifs [36], using quantitative measures. We found that all three motifs reported from the predicted CRMs matched the *Dorsal *PWM (Figure 2), while the motifs discovered in the entire data set did not match any known motif significantly (see Materials and methods). *Dorsal *is one of the key regulators of the neuroectoderm development gene battery.

This result is an example of how CRM finding can precede motif-finding in *cis*-regulatory analysis, rather than rely on prior knowledge or discovery of the motifs. A thorough investigation of this claim, using many motif finding methods and all data sets, will be performed in future work.

## Discussion

We have made certain choices in our benchmark construction and evaluation that were guided by an intent to keep the procedure simple. The choice of requiring every program to report equal length windows, even though the known (planted) modules are not equally long, is an example. The work of Tompa *et al*. [18] on evaluating motif-finding programs highlighted the difficulties of comparing programs that make different 'amounts' of prediction. In this first comprehensive evaluation of a new formulation of CRM prediction, we chose to avoid such complications by ensuring that the sensitivity and specificity are always identical. Admittedly, some programs, such as CisModule, have the additional capability to predict modules of varying lengths, while others, like CSam, D2Z-set or MCD, currently have no principled way to do this. In the future, evaluation procedures may attempt to also assess programs for this ability.

Each of the 33 data sets in our benchmark is constructed from CRMs that drive expression in the same tissue (and/or stage of development), but not necessarily in the same cells. As such, all genes in a data set may not 'truly' belong to the same gene battery, leading to some 'noise' in definition of the data set. This may explain, at least partly, why some data sets do not show significant prediction accuracy with any method - the members do not actually constitute a gene battery.

We have proposed two new CRM prediction methods (CSam and D2Z-set), whose most important aspect is that they do not rely on known motifs or the ability to discover precise motifs computationally. This is inspired by our cautious skepticism about the accuracy of *ab initio *motif finding as of today. This is not meant to suggest that there is no 'motif-finding component' to these algorithms at all. Indeed, both algorithms look at frequencies of short words, as do many motif-finding programs, but without the usual objective of finding the most specific (biochemically accurate) characterization of the transcription factor's binding sites. Instead, the algorithms are guided by statistical effects of present binding sites on the full complement of short words in the CRMs.

The PFRSampler algorithm of Grad *et al*. [16] (which we have re-implemented as 'MCD') also subscribes to the above general principle. However, one of its drawbacks is that the sequence composition is captured in terms of raw counts of short words, during construction of the Markov chain. It has been shown that raw counts of different short words (of same length) follow different statistical distributions (asymptotically normal, with different means and variances). Thus, an approach that weighs all words equally in their contribution to the overall characterization of the CRM is subject to biases. CSam addresses this issue by assessing the statistical significance of each short word appropriately before counting overrepresented motifs.

An interesting aspect of our work is to show that CRM prediction does not have to be the step that follows motif-prediction (as in Ahab, Stubb) or goes hand-in-hand with it (as in CisModule, EMCModule); in fact, it can precede motif-finding as a signal-boosting step where the control regions are trimmed down. Given a gene battery and the task of finding motifs in its control regions, one daunting challenge is the sheer size of these regions, which may be tens of kilobases long in metazoan genomes. Motif finding is usually an intractable problem for such input sizes. Thus, predicting CRMs may be a manageable first step, which if followed by motif-finding inside the CRMs may lead to accurate binding site prediction. We provide an example of this in the previous section. This claim, however, needs a thorough investigation, using different motif-finding pipelines and many more data sets; this is beyond the scope of the current paper.

## Conclusion

We have focused on a relatively novel paradigm of computational module discovery, the 'gene battery CRM discovery' problem. This addresses the need to take computational approaches to the more challenging realm of uncharted regulatory networks, in order to have greater applicability. We have presented a benchmark of 33 data sets, built from real *Drosophila *modules and genomic sequences, far more comprehensive than the 2 or 3 data sets available today. The data sets are designed to be realistic, yet allow for simple evaluation of CRM prediction algorithms. The benchmark is available publicly, along with necessary code for its use. This will be a valuable resource for the community, encouraging new development and evaluations for this very important problem. We have also presented two novel methods for *ab initio *CRM discovery, and demonstrated that they perform significantly well on a majority of the benchmark data sets. These new methods themselves represent a novel paradigm of *cis*-regulatory analysis, where accurate motif discovery is not called upon for module discovery.

## Materials and methods

### Construction of benchmarks

Each CRM was planted at a randomly chosen position in a background sequence of length nine times its own length, thus creating a semi-real "control region" of length ten times that of the CRM. The background sequence was constructed by concatenating 1,000 bp non-coding sub-sequences of the *D. melanogaster *genome, each sub-sequence having G/C content similar to the '9×' length region around the CRM.

### Stubb runs

Stubb was run with a set of experimentally validated motifs known to play a regulatory role in the fruitfly. The 53 PWMs from FlyREG that were used are listed in Additional data file 1. The window length input to Stubb was the average size for a module in a given data set. The background model used was a 'global background' of Markov order 0, trained on the entire data set.

### Post-processing of CisModule output

CisModule can output multiple module predictions of various lengths in the same input sequence. It computes a posterior probability for every position being inside a module, and uses a threshold of 0.5 on this posterior probability to predict modules. In our evaluations, the original output of CisModule led to a very large number of positions being predicted as modules and, thus, to very high sensitivity and very low specificity. For consistency of evaluations, we post-processed CisModule output as follows. Suppose *L*_*c *_is the desired module length, computed as the average module length in a data set. From the output of CisModule, we chose the contiguous *L*_*c*_-length window with the maximum sum of posterior probability scores.

### CSam algorithm

Let us define a 'motif' as any string of length *k *over the alphabet {A,C,G,T,R,Y,W,S}, such that, at most, *d *characters are 'degenerate' (R,Y,W, or S). The set of all motifs for particular values of *k *and *d *is called the (*k*,*d*) motif space. A motif is said to be σ-significant in a set of sequences *R *if its number of occurrences in *R *is σ standard deviations above the number expected by chance, assuming a suitable null model of sequence generation. The null model we will assume is a third order Markov chain over the alphabet {A,C,G,T}, with suitable trained parameters.

The 'motif count score' for a set of sequences *R*, denoted by *MCS*_*k*,*d,σ *_(*R*), is the number of motifs from the (*k*,*d*) motif space that are σ-significant in *R*. For given values of *k*,*d*,σ, the program YMF [35] can compute this score efficiently. We henceforth drop the suffix and denote the motif count score as *MCS*(*R*).

**Figure 3:**
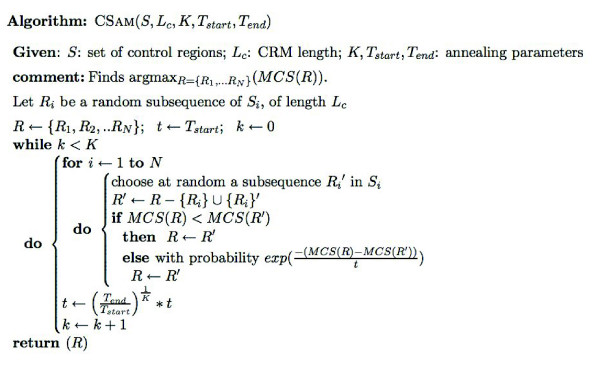
Pseudo-code for the CSam algorithm.

Given a set of control regions *S *= {*S*_1_,...*S*_*N*_} of *N *co-regulated genes, the CSam algorithm tries to find a set *R *= {*R*_1_,...*R*_*N*_} of sub-sequences of length *L*_*c*_, exactly one *R*_*i *_in each *S*_*i*_, such that *MCS*(*R*) is maximized. The implementation uses a simulated annealing search to sample the space of all possible locations for CRMs in *S*, where the energy function of the current sample *R *is *E*(*R*) = -*MCS*(*R*). The transition probability from a sample *R *to a 'neighboring' sample *R*' is defined by a Metropolis-Hastings strategy, and is equal to min$\left(1,exp(\frac{-(E(R')-E(R))}{t})\right)$, with *t *being the current temperature in the sampler. The algorithm starts by initializing the current set of CRMs *R *to random subsequences of length *L*_*c*_, one in each sequence from *S*. In each sampling step, an element *R*_*i*_∈*R *is chosen (in a round robin fashion), and replaced with another subsequence *R*_*i*_', chosen at random from the same *S*_*i*_, to obtain a new solution *R*'=*R*-{*R*_*i*_}∪{*R*_*i*_'}. (All other *R*_*j*_∈*R*, for *j*≠*i*, are kept fixed in this step.) After re-sampling each *R*_*i*_∈*R* in this way, a new temperature is reached by following a proportional cooling schedule. The pseudocode for CSam is shown in Figure 3.

The algorithm remembers the best scoring solution seen during the sampling, and reports this upon termination. An early termination is possible if the score does not change over some fixed number of iterations. Also, to ensure that the algorithm does not get stuck in a local optimum, we perform several random restarts.

The *MCS *score is calculated using YMF routines [35], with parameters *k *= 6 and *d *= 1, which enumerate motifs of length 6 with, at most, one degenerate character, and their associated significance values (z-scores). Counting motifs with z-score greater or equal to σ = 3 results in the *MCS *score. While actual binding sites (and motifs) are typically longer than 6 bp, our choice of *k *= 6 attempts to capture the significant 'core'(s) of these sites.

### D2Z-set algorithm

Let *N*_*i*_(*w*) be the count of word *w *in sequence *S*_*i*_. The *D*2 score between two sequences *S*_*i *_and *S*_*j *_is defined as:

(2) $$D2(S_{i},S_{j})=\int_{w}N_{i}(w)N_{j}(w)$$

where the sum is over all 4^*k *^words of length *k*. Note that this definition is equivalent to the number of *k*-mer matches between *S*_*i *_and *S*_*j*_. It is expected that two sequences with the same regulatory elements will share a larger number of *k*-mers than two random unrelated sequences. The probability distribution of the *D*2 score is dependent on the background models assumed to have generated each of the two sequences. Therefore, for comparison across different pairs of sequences, the *D*2 score needs to be 'normalized' suitably. The mean *E*(*D*2) and standard deviation σ(*D*2) of the D2 score can be computed as in [17], giving us a z-score defined as:

(3) $$D2Z(S_{i},S_{j})=\frac{D2(S_{i},S_{j})-E(D2)}{\sigma (D2)}$$

The mean *E*(*D*2) and standard deviation σ(*D*2) are calculated under a null model where each of the two compared sequences is generated by a separate zeroth order Markov chain. In our tests, we used *k *= 5, based on recommendations of [17] and the fact that this score only counts exact matches between words. To score a given set of putative CRMs, we partition the set at random into two halves, and concatenate the sequences in each half. The D2Z score between these two concatenated sequences is then computed, measuring the similarity between the two original subsets. This is repeated a fixed number of times and the average D2Z score is computed, giving us the 'D2Z-set' score of the candidate set. (Each repetition involves recalculation of the mean and standard deviation used in equation 3, since the background or null model has changed.) We use simulated annealing to search for the set of windows with maximum D2Z-set score. The search algorithm is identical to that of CSam, except that the energy function is now given by *E*(*R*) = -'D2Z-set'(*R*).

### MCD algorithm

The MCD algorithm uses the scoring function first introduced by Grad *et al*. [16]. The score rewards a set of sequences whose *k*-mer compositions are most similar to each other and different from other sequences. For this purpose, a fifth order Markov chain (called *model*+) is trained on an entire set of given candidate CRMs, and another Markov chain (called *model*-) is trained on the remaining portions of the input control regions. Each putative CRM sequence is scored by the log-odds of *model*+ to *model*-, as given by equation 4:

(4) $$MCD(s)=log\frac{P(s|model+)}{P(s|model-)}=\int_{i=1}^{L}log\frac{a_{s_{i-1}s_{i}}^{+}}{a_{s_{i-1}s_{i}}^{-}}$$

where $a_{s_{i}s_{j}}^{+}$ is the transition probability from s_*i *_to the last character of *s*_*j *_in *model*+, and $a_{s_{i}s_{j}}^{-}$ is similarly defined for *model*-. The score of the set of candidate CRMs is the sum of the above log-odds score over each candidate. Due to the limited size of the sequence training data, it is important to smooth the transition probabilities of the Markov chains. Therefore, the counts for transitions from 5-mer *s *to character *d *(required to train the Markov chain) are smoothed by adding the counts of transitions to *d *from all 5-mers that are within one mismatch from *s*. These smoothing terms are added after 'weighting' them by a fixed small value. Our experiments are reported with a smoothing factor of 0.1. Having thus defined the score of a candidate set of CRMs, the MCD method uses simulated annealing to search for the maximally scoring set in the control regions.

### Variations on new algorithms

The algorithms CSam, D2Z-set, and MCD needed some experimentation to decide the correct score parameters, search strategy, and so on. We therefore compared different versions of each algorithm on synthetic data sets, in order to choose the optimal version. To construct a synthetic data set, we began with randomly chosen PWMs (of specificity similar to real PWMs), and used the probabilistic model of Stubb to generate synthetic CRMs that contained randomly sampled binding sites for these PWMs. The synthetic CRM was then planted in randomly generated background sequence to give us a synthetic control region. Many such control regions defined a data set. The evaluation of a prediction method on such data sets was done in the same manner as for the benchmark data sets.

We compared the simulated annealing and Gibbs sampling strategies for CSam and found little difference. The default D2Z method was compared to its variants, where word length *k *was changed to 6, or word counting was done on both strands, or Gibbs sampling was used instead of simulated annealing. The default version performed best in these tests. For the MCD method, we experimented with weights (smoothing factor) of 0.1 and 0.25 and did not notice significant differences.

### Running time

The running times for each data set (on an Intel Xeon workstation running Linux) are shown in Table S4 in Additional data file 1. In general, we see that D2Z-set and MCD have lower running times than CSam, but for the larger data sets (over 40 sequences), CSam is often faster than D2Z-set and MCD. CisModule is always the most computationally expensive method in our tests. This is intentional, since the number of sampling iterations of each of the other methods was configured to achieve this.

### Motif finding

The motif discovery mentioned in the Results section predicted motifs as consensus strings, which were converted to PWM representation, and compared to 53 motifs from the FlyREG database. To compare any two given motifs, we used their relative entropy as the measure of difference, and computed empirical *p*-values of this measure: each of the original motifs was shuffled to produce 1,000 randomized versions, each shuffled version was compared to the original version of the other motif, and a *p*-value was obtained. We declared a predicted motif to match a known motif if their relative entropy was less than 0.5 bits per column, and both computed *p*-values were below 0.005.

## Abbreviations

CRM, *cis*-regulatory module; MCD, Markov chain discrimination; PWM, position weight matrix.

## Authors' contributions

SS and MH designed the overall project and creation of data sets, SS and AI designed algorithms, AI performed implementations and experiments, and AI, MH and SS wrote the paper.

## Additional data files

The following additional data are available. Additional data file 1 is a PDF file with Tables S1-S7, and the URL for downloading complete benchmark data sets and evaluation script.

## Supplementary Material

Additional data file 1 Supplementary Tables S1-S7, and a URL for downloading complete benchmark data sets and evaluation script. Click here for file
