# Supplementary material for: Computational discovery of cis-regulatory modules in Drosophila without prior knowledge of motifs
Source: Genome Biol. 2008 Jan 28;9(1):R22. doi: 10.1186/gb-2008-9-1-r22 (PMC2395258; doi:10.1186/gb-2008-9-1-r22)
Supplement: Additional data file 1 — Supplementary Tables S1-S7, and a URL for downloading complete benchmark data sets and evaluation script. [file gb-2008-9-1-r22-S1.pdf]

Supplementary Materials: Computational discovery of cis-regulatory modules in *Drosophila*, without prior knowledge of motifs

Andra Ivan , Marc Halfon and Saurabh Sinha

Online supplementary materials available at: http://veda.cs.uiuc.edu/crmfinding/
This includes:

1. Set of PWMs used by Stubb.
2. Benchmark data sets with evaluation code.

Table S1: Performance of MCD and CisModule on 33 datasets in our benchmark. Column 2 shows the number of sequences in a data set, the total sequence length, and the maximum sensitivity possible. Columns 3 and 4 show the sensitivity and its empirical p-value for MCD and CisModule respectively.

**Data set**	**#Seq, Length, Max. Sens.**	**MCD P-val (Sens)**	**CisModule P-val (Sens)**
mapping3.adult	34, 254800, 0.71	0.44 (0.10)	0.21 (0.12)
mapping1.adult mesoderm	5, 28085, 0.76	0.16 (0.20)	1.00 (0.00)
mapping1.amnioserosa	7, 49635, 0.84	0.13 (0.20)	0.78 (0.01)
mapping1.blastoderm	77, 698840, 0.77	0.95 (0.06)	0.79 (0.07)
mapping1.cardiac mesoderm	8, 42979, 0.76	0.08 (0.22)	0.74 (0.04)
mapping1.cns	34, 352108, 0.80	0.25 (0.12)	0.27 (0.12)
mapping1.dorsal ectoderm	8, 67490, 0.77	0.58 (0.07)	0.69 (0.05)
mapping1.ectoderm	37, 311000, 0.72	0.69 (0.08)	0.56 (0.09)
mapping2.ectoderm	51, 416473, 0.74	0.19 (0.12)	0.66 (0.08)
mapping1.endoderm	16, 92723, 0.82	0.69 (0.06)	0.07 (0.18)
mapping1.eye	6, 49494, 0.70	0.54 (0.07)	0.10 (0.22)
mapping2.eye	18, 156531, 0.69	0.92 (0.03)	0.51 (0.09)
mapping1.fat body	5, 22831, 0.93	1.00 (0.00)	1.00 (0.00)
mapping1.female gonad	10, 44269, 0.62	0.74 (0.04)	0.81 (0.03)
mapping1.glia	7, 63008, 0.82	**0.01** (0.36)	0.68 (0.05)
mapping1.imaginal disc	47, 441597, 0.77	0.47 (0.10)	0.54 (0.09)
mapping2.imaginal disc	12, 149915, 0.80	0.19 (0.16)	0.82 (0.04)
mapping3.larva	69, 616635, 0.76	0.61 (0.08)	0.16 (0.12)
mapping1.male gonad	8, 69044, 0.85	0.72 (0.03)	0.31 (0.13)
mapping1.malpighian tubules	4, 31338, 0.81	0.42 (0.10)	1.00 (0.00)
mapping1.mesectoderm	5, 45712, 0.83	0.23 (0.16)	0.63 (0.03)
mapping1.mesoderm	16, 87140, 0.72	0.35 (0.11)	**0.01** (0.23)
mapping2.mesoderm	45, 233441, 0.75	0.07 (0.14)	0.94 (0.04)
mapping1.neuroectoderm	7, 40315, 0.80	0.17 (0.17)	0.14 (0.18)
mapping2.neuronal	54, 534081, 0.78	0.77 (0.07)	0.92 (0.05)
mapping1.pns	24, 234532, 0.78	**0.01** (0.23)	0.40 (0.10)
mapping2.reproductive system	21, 154400, 0.69	0.25 (0.12)	0.62 (0.07)
mapping1.salivary gland	6, 47232, 0.74	0.70 (0.03)	0.29 (0.14)
mapping1.somatic muscle	12, 86317, 0.79	0.81 (0.04)	0.16 (0.15)
mapping1.tracheal system	9, 111351, 0.85	0.77 (0.04)	1.00 (0.00)
mapping1.ventral ectoderm	12, 84154, 0.77	0.25 (0.14)	0.62 (0.07)
mapping1.visceral mesoderm	12, 54278, 0.77	**0.00** (0.37)	0.29 (0.13)

Table S2: Score p-values for MCD, D2Z-set, and CSam on all data sets.

**Data Set**	**No. Seq**	**MCD**	**D2Z-set**	**CSam**
mapping3.adult	34	0.44	0.72	0.15
mapping1.adult mesoderm	5	0.16	0.11	0.51
mapping1.amnioserosa	7	0.13	0.34	0.09
mapping1.blastoderm	77	0.95	0.10	**0.00**
mapping1.cardiac mesoderm	8	0.08	**0.03**	0.12
mapping1.cns	34	0.25	**0.01**	**0.02**
mapping1.dorsal ectoderm	8	0.58	0.88	0.08
mapping1.ectoderm	37	0.69	**0.00**	**0.00**
mapping2.ectoderm	51	0.19	**0.05**	**0.00**
mapping1.endoderm	16	0.69	0.31	**0.01**
mapping1.eye	6	0.54	0.48	**0.02**
mapping2.eye	18	0.92	0.68	0.88
mapping1.fat body	5	1.00	1.00	0.45
mapping1.female gonad	10	0.74	0.97	0.86
mapping1.glia	7	**0.01**	0.16	0.21
mapping1.imaginal disc	47	0.47	**0.00**	0.24
mapping2.imaginal disc	12	0.19	0.12	0.33
mapping3.larva	69	0.61	**0.02**	**0.00**
mapping1.male gonad	8	0.72	0.46	0.15
mapping1.malpighian tubules	4	0.42	1.00	0.30
mapping1.mesectoderm	5	0.23	0.43	**0.00**
mapping1.mesoderm	16	0.35	0.09	0.22
mapping2.mesoderm	45	0.07	**0.00**	**0.02**
mapping1.neuroectoderm	7	0.17	1.00	**0.00**
mapping2.neuronal	54	0.77	**0.00**	**0.00**
mapping1.pns	24	**0.01**	0.07	**0.01**
mapping2.reproductive system	21	0.25	0.34	0.24
mapping1.salivary gland	6	0.70	1.00	0.36
mapping1.somatic muscle	12	0.81	0.05	**0.01**
mapping1.tracheal system	9	0.77	0.21	0.18
mapping1.ventral ectoderm	12	0.25	0.32	**0.01**
mapping1.visceral mesoderm	12	**0.00**	0.32	**0.01**
mapping2.wing	33	0.25	**0.00**	**0.00**

Table S3: Sensitivity p-values for CSam run on data sets with CRM size to total size in the ratio of 1:10 (X10) and 1:5 (X5) respectively. Column 2 shows the number of sequences in a data set, the total sequence length, and the maximum sensitivity possible. Columns 3 and 4 show the sensitivity (Sens) and its p-value (P) on each data set for X10 and X5 versions of the data set, respectively.

**Set**	**No. Seq, Length, Max. sens.**	**CSam -X10 P | Sens.**	**CSam -X5 P | Sens.**
mapping1.adult	34, 254800, 0.71	0.15 (0.13)	**0.01** (0.29)
mapping1.adult mesoderm	5, 28085, 0.76	0.51 (0.05)	0.18 (0.28)
mapping1.amnioserosa	7, 49635, 0.84	0.09 (0.23)	0.16 (0.29)
mapping1.blastoderm	77, 698840, 0.76	**0.00** (0.26)	**0.00** (0.35)
mapping1.cardiac mesoderm	8, 42979, 0.76	0.12 (0.19)	**0.03** (0.37)
mapping1.cns	34, 352108, 0.80	**0.02** (0.18)	**0.00** (0.36)
mapping1.dorsal ectoderm	8, 67490, 0.77	0.08 (0.22)	0.78 (0.11)
mapping1.ectoderm	37, 311000, 0.72	**0.00** (0.21)	**0.05** (0.26)
mapping2.ectoderm	51, 416473, 0.73	**0.00** (0.23)	**0.00** (0.32)
mapping1.endoderm	16, 92723, 0.82	**0.01** (0.26)	**0.00** (0.40)
mapping1.eye	6, 49494, 0.70	**0.02** (0.32)	0.48 (0.17)
mapping2.eye	18, 156531, 0.69	0.88 (0.04)	0.26 (0.20)
mapping1.fat body	5, 22831, 0.93	0.45 (0.09)	**0.01** (0.53)
mapping1.female gonad	10, 44269, 0.62	0.86 (0.02)	0.63 (0.14)
mapping1.glia	7, 63008, 0.82	0.21 (0.17)	0.10 (0.32)
mapping1.imaginal disc	47, 441597, 0.76	0.24 (0.12)	**0.00** (0.35)
mapping2.imaginal disc	12, 149915, 0.80	0.33 (0.12)	**0.02** (0.36)
mapping3.larva	69, 616635, 0.76	**0.00** (0.18)	**0.00** (0.31)
mapping1.male gonad	8, 69044, 0.85	0.15 (0.18)	0.16 (0.27)
mapping1.malpighian tubules	4, 31338, 0.81	0.30 (0.16)	1.00 (0.00)
mapping1.mesectoderm	5, 45712, 0.83	**0.00** (0.46)	0.29 (0.24)
mapping1.mesoderm	16, 87140, 0.72	0.22 (0.13)	**0.00** (0.42)
mapping2.mesoderm	45, 233441, 0.75	**0.02** (0.16)	**0.00** (0.37)
mapping1.neuroectoderm	7, 40315, 0.80	**0.00** (0.51)	**0.00** (0.53)
mapping2.neuronal	54, 534081, 0.78	**0.00** (0.26)	**0.00** (0.36)
mapping1.pns	24, 234532, 0.78	**0.01** (0.21)	**0.00** (0.45)
mapping2.reproductive system	21, 154400, 0.69	0.24 (0.12)	**0.03** (0.28)
mapping1.salivary gland	6, 47232, 0.74	0.36 (0.11)	0.71 (0.11)
mapping1.somatic muscle	12, 86317, 0.79	**0.01** (0.28)	**0.01** (0.37)
mapping1.tracheal system	9, 111351, 0.85	0.18 (0.17)	0.38 (0.22)
mapping1.ventral ectoderm	12, 84154, 0.77	**0.01** (0.27)	0.15 (0.28)
mapping1.visceral mesoderm	12, 54278, 0.77	**0.01** (0.28)	**0.00** (0.42)
mapping2.wing	33, 340094, 0.78	**0.00** (0.22)	**0.00** (0.39)

Table S4: Running times (hours, minutes) for D2Z, MCD, CSam, CisModule on each data set.

**Set**	**D2Z**	**MCD**	**CSam**	**CisModule**
mapping3.adult	4h 18m	4h 3m	9h 28m	9h 34m
mapping1.adult mesoderm	0h 7m	0h 4m	1h 17m	1h 10m
mapping1.amnioserosa	0h 19m	0h 10m	1h 5m	2h
mapping1.blastoderm	27h 37m	26h 10m	15h 26m	26h 35m
mapping1.cardiac mesoderm	0h 21m	0h 10m	1h 10m	1h 41m
mapping1.cns	4h 18m	5h 29m	6h 1m	14h 47m
mapping1.dorsal ectoderm	0h 26m	0h 16m	1h 14m	2h 49m
mapping1.ectoderm	5h 13m	5h 20m	6h 28m	12h 11m
mapping2.ectoderm	10h 51m	9h 44m	9h 17m	17h 25m
mapping1.endoderm	1h 10m	0h 42m	2h 34m	3h 28m
mapping1.eye	0h 19m	0h 10m	1h 49m	2h 4m
mapping2.eye	1h 24m	1h 23m	2h 49m	5h 20m
mapping1.fat body	0h 7m	0h 3m	0h 44m	0h 49m
mapping1.female gonad	0h 31m	0h 13m	1h 28m	1h 42m
mapping1.glia	0h 22m	0h 13m	1h 53m	2h 19m
mapping1.imaginal disc	8h 53m	9h 26m	8h 22m	16h 50m
mapping2.imaginal disc	0h 46m	0h 52m	1h 56m	5h 39m
mapping3.larva	22h 6m	19h 38m	12h 49m	23h 44m
mapping1.male gonad	0h 26m	0h 16m	1h 14m	2h 56m
mapping1.malpighian tubules	0h 7m	0h 4m	0h 39m	1h 9m
mapping1.mesectoderm	0h 18m	0h 7m	0h 47m	1h 44m
mapping1.mesoderm	1h 10m	0h 40m	2h 30m	3h 16m
mapping2.mesoderm	7h 39m	4h 58m	7h 6m	8h 32m
mapping1.neuroectoderm	0h 18m	0h 9m	1h 1m	1h 32m
mapping2.neuronal	12h 38m	13h 58m	10h 24m	20h 5m
mapping1.pns	2h 14m	2h 37m	6h 46m	9h 16m
mapping2.reproductive system	1h 48m	1h 34m	3h 32m	6h 8m
mapping1.salivary gland	0h 19m	0h 8m	1h 32m	1h 49m
mapping1.somatic muscle	0h 45m	0h 29m	1h 49m	3h 31m
mapping1.tracheal system	0h 31m	0h 30m	1h 28m	3h 55m
mapping1.ventral ectoderm	0h 46m	0h 30m	3h 12m	3h 32m
mapping1.visceral mesoderm	0h 45m	0h 19m	1h 51m	2h 14m
mapping2.wing	3h 59m	5h 6m	5h 44m	12h 29m

Table S5: Performance of Stubb, CSam, D2z-set, CisModule and MCD, when all tests are performed with an input CRM length of 750 bp. Columns 2-6 show the sensitivity and its empirical p-value for each method. Significant performance values (p-value ≤ 0.05) are shown in boldface.

**Data set**	**Stubb Pval (Sens)**	**CSam Pval (Sens)**	**D2z-set Pval (Sens)**	**CisModule Pval (Sens)**	**MCD Pval (Sens)**
mapping3 adult	**0.01 (0.20)**	0.11 (0.14)	0.84 (0.06)	0.64 (0.08)	0.91 (0.05)
mapping1 adult mesoderm	0.51 (0.05)	0.15 (0.25)	0.10 (0.29)	0.16 (0.19)	0.18 (0.23)
mapping1 amnioserosa	0.33 (0.13)	0.20 (0.18)	1.00 (0.00)	0.28 (0.14)	0.65 (0.06)
mapping1 blastoderm	**0.00 (0.31)**	**0.00 (0.25)**	0.29 (0.09)	0.91 (0.06)	0.96 (0.04)
mapping1 cardiac mesoderm	0.14 (0.19)	**0.05 (0.32)**	**0.04 (0.32)**	0.12 (0.20)	**0.02 (0.38)**
mapping1 cns	0.64 (0.08)	0.09 (0.11)	**0.02 (0.14)**	0.81 (0.06)	0.17 (0.10)
mapping1 dorsal ectoderm	0.14 (0.19)	0.42 (0.09)	0.77 (0.03)	0.45 (0.10)	0.72 (0.04)
mapping1 ectoderm	0.09 (0.15)	**0.01 (0.17)**	**0.01 (0.18)**	0.55 (0.09)	0.54 (0.08)
mapping2 ectoderm	**0.05 (0.15)**	**0.00 (0.21)**	**0.04 (0.14)**	0.66 (0.08)	0.19 (0.11)
mapping1 endoderm	**0.01 (0.26)**	**0.00 (0.34)**	0.20 (0.17)	**0.04 (0.20)**	0.69 (0.08)
mapping2 eye	0.30 (0.12)	0.55 (0.08)	0.66 (0.06)	0.54 (0.09)	0.72 (0.06)
mapping1 eye	1.00 (0.00)	0.30 (0.12)	0.46 (0.08)	0.27 (0.14)	0.41 (0.10)
mapping1 fat body	1.00 (0.00)	0.06 (0.40)	0.74 (0.01)	**0.02 (0.37)**	0.28 (0.22)
mapping1 female gonad	**0.00 (0.44)**	0.57 (0.11)	0.71 (0.08)	0.32 (0.11)	0.83 (0.06)
mapping1 glia	0.79 (0.01)	0.44 (0.08)	0.32 (0.11)	0.47 (0.09)	**0.00 (0.40)**
mapping1 imaginal disc	0.73 (0.08)	**0.00 (0.16)**	0.06 (0.12)	0.54 (0.09)	0.17 (0.10)
mapping2 imaginal disc	0.73 (0.05)	0.48 (0.06)	0.56 (0.05)	0.73 (0.05)	0.23 (0.09)
mapping3 larva	0.19 (0.11)	**0.00 (0.16)**	**0.00 (0.15)**	0.72 (0.07)	0.98 (0.04)
mapping1 male gonad	0.38 (0.12)	0.61 (0.05)	0.40 (0.10)	0.35 (0.12)	0.76 (0.03)
mapping1 malpighian tubules	0.13 (0.24)	1.00 (0.00)	1.00 (0.00)	0.44 (0.09)	0.42 (0.10)
mapping1 mesectoderm	0.33 (0.13)	1.00 (0.00)	0.45 (0.08)	0.44 (0.10)	0.19 (0.16)
mapping1 mesoderm	**0.01 (0.25)**	0.19 (0.18)	0.15 (0.19)	0.36 (0.10)	0.98 (0.01)
mapping2 mesoderm	**0.00 (0.25)**	**0.00 (0.37)**	**0.00 (0.37)**	**0.03 (0.16)**	0.24 (0.15)
mapping1 neuroectoderm	**0.00 (0.38)**	**0.03 (0.34)**	0.83 (0.00)	**0.05 (0.25)**	0.20 (0.20)
mapping2 neuronal	0.49 (0.09)	**0.00 (0.22)**	**0.00 (0.15)**	0.66 (0.08)	0.90 (0.04)
mapping1 pns	**0.05 (0.17)**	**0.00 (0.26)**	0.49 (0.07)	0.76 (0.06)	**0.01 (0.18)**
mapping2 reproductive system	0.17 (0.13)	0.12 (0.15)	0.23 (0.12)	0.37 (0.10)	0.26 (0.12)
mapping1 salivary gland	0.71 (0.03)	0.35 (0.11)	1.00 (0.00)	0.62 (0.06)	0.65 (0.04)
mapping1 somatic muscle	0.29 (0.12)	**0.01 (0.28)**	0.06 (0.21)	0.14 (0.16)	0.68 (0.06)
mapping1 tracheal system	0.75 (0.04)	0.64 (0.04)	**0.00 (0.26)**	0.75 (0.04)	0.06 (0.15)
mapping1 ventral ectoderm	**0.00 (0.49)**	0.37 (0.12)	0.29 (0.14)	0.60 (0.08)	**0.03 (0.25)**
mapping1 visceral mesoderm	0.25 (0.14)	**0.01 (0.41)**	0.39 (0.17)	0.07 (0.20)	0.75 (0.09)
mapping2 wing	0.32 (0.11)	**0.00 (0.18)**	**0.01 (0.15)**	0.61 (0.08)	0.56 (0.06)

Table S6: Performance of Stubb, CSam, D2z-set, CisModule and MCD, on "native" data sets. Significant performance values (p-value ≤ 0.05) are shown in boldface. Data sets were constructed as follows: (i) For each CRM, extract its flanking region in the genome; the length of this control region is chosen to be 9 times the length of the CRM. (ii) If the control region of one CRM intersects that of another, or intersects another CRM, "shift" either the first or the second control region, such that there are no overlapping control regions in the same data set. (This implies that each resulting control region has one CRM within it.) (iii) If a control region overlaps with a gene, the gene portion of the control region is masked, thereby reducing the size of the control region. Out of the original total of 718 sequences, 206 were altered in this way.

**Data set**	**Stubb P-val (Sens)**	**CSam P-val (Sens)**	**D2z-set P-val (Sens)**	**CisModule P-val (Sens)**	**MCD P-val (Sens)**
mapping3 adult	0.29 (0.12)	0.08 (0.16)	0.41 (0.11)	0.29 (0.12)	0.30 (0.12)
mapping1 adult mesoderm	0.74 (0.02)	**0.05 (0.30)**	0.53 (0.04)	0.11 (0.23)	0.74 (0.03)
mapping1 amnioserosa	0.74 (0.02)	0.15 (0.19)	0.08 (0.23)	0.33 (0.12)	1.00 (0.00)
mapping1 blastoderm	**0.00 (0.24)**	**0.04 (0.15)**	0.09 (0.14)	0.29 (0.12)	**0.01 (0.17)**
mapping1 cardiac mesoderm	0.60 (0.07)	0.15 (0.18)	**0.00 (0.35)**	0.28 (0.13)	0.77 (0.03)
mapping1 cns	0.81 (0.07)	0.99 (0.03)	0.11 (0.16)	0.56 (0.10)	0.33 (0.13)
mapping1 dorsal ectoderm	**0.00 (0.38)**	0.42 (0.11)	0.30 (0.14)	0.22 (0.16)	0.26 (0.15)
mapping1 ectoderm	**0.01 (0.19)**	0.08 (0.16)	0.19 (0.13)	0.30 (0.12)	0.65 (0.09)
mapping2 ectoderm	**0.00 (0.22)**	**0.01 (0.19)**	0.19 (0.13)	0.51 (0.10)	0.62 (0.09)
mapping1 endoderm	0.15 (0.16)	0.15 (0.16)	0.55 (0.09)	0.47 (0.10)	0.19 (0.15)
mapping1 eye	0.25 (0.17)	0.56 (0.08)	1.00 (0.00)	0.39 (0.12)	0.14 (0.21)
mapping2 eye	0.34 (0.14)	0.80 (0.07)	0.08 (0.20)	0.57 (0.10)	0.78 (0.07)
mapping1 fat body	0.42 (0.11)	1.00 (0.00)	1.00 (0.00)	0.20 (0.18)	0.64 (0.02)
mapping1 female gonad	0.39 (0.10)	0.24 (0.13)	0.07 (0.19)	0.53 (0.07)	0.32 (0.11)
mapping1 glia	0.71 (0.05)	0.11 (0.23)	0.14 (0.22)	0.36 (0.14)	0.73 (0.04)
mapping1 imaginal disc	0.87 (0.07)	**0.00 (0.21)**	**0.03 (0.18)**	0.45 (0.11)	**0.00 (0.21)**
mapping2 imaginal disc	0.97 (0.01)	0.41 (0.14)	0.16 (0.21)	0.29 (0.17)	0.65 (0.09)
mapping3 larva	0.70 (0.09)	0.53 (0.11)	0.15 (0.14)	0.52 (0.11)	0.93 (0.07)
mapping1 male gonad	0.38 (0.12)	0.10 (0.21)	0.82 (0.02)	0.10 (0.21)	0.25 (0.15)
mapping1 malpighian tubules	0.06 (0.32)	0.59 (0.02)	1.00 (0.00)	0.54 (0.05)	1.00 (0.00)
mapping1 mesectoderm	0.24 (0.18)	0.10 (0.26)	1.00 (0.00)	0.47 (0.10)	1.00 (0.00)
mapping1 mesoderm	**0.00 (0.28)**	**0.05 (0.20)**	0.07 (0.19)	0.69 (0.07)	**0.04 (0.20)**
mapping2 mesoderm	0.06 (0.15)	**0.02 (0.17)**	0.11 (0.14)	0.19 (0.12)	0.06 (0.15)
mapping1 neuroectoderm	**0.02 (0.31)**	0.28 (0.15)	0.19 (0.18)	0.46 (0.10)	0.09 (0.23)
mapping2 neuronal	0.48 (0.11)	0.21 (0.13)	**0.02 (0.18)**	0.60 (0.10)	**0.04 (0.17)**
mapping1 pns	**0.03 (0.21)**	**0.01 (0.24)**	0.65 (0.09)	0.19 (0.15)	0.13 (0.16)
mapping2 reproductive system	0.15 (0.15)	0.82 (0.05)	0.29 (0.12)	0.26 (0.13)	0.82 (0.05)
mapping1 salivary gland	0.26 (0.16)	0.42 (0.11)	0.27 (0.16)	0.30 (0.15)	1.00 (0.00)
mapping1 somatic muscle	0.61 (0.08)	0.06 (0.22)	0.94 (0.01)	0.70 (0.06)	0.23 (0.15)
mapping1 tracheal system	0.15 (0.21)	0.39 (0.14)	0.28 (0.17)	0.36 (0.14)	**0.03 (0.31)**
mapping1 ventral ectoderm	0.14 (0.18)	0.61 (0.08)	0.56 (0.09)	0.21 (0.16)	0.24 (0.15)
mapping1 visceral mesoderm	0.21 (0.14)	**0.01 (0.26)**	0.25 (0.13)	0.38 (0.11)	0.17 (0.16)
mapping2 wing	0.41 (0.12)	0.22 (0.14)	**0.01 (0.21)**	0.20 (0.15)	0.12 (0.16)

Table S7: CRM-level sensitivity of Stubb, CSam, D2z-set, CisModule and MCD, on “native” data sets. Columns 3 - 7 show the fraction (and number) of CRMs in a data set that were “hits”. Best CRM-level sensitivity for each data set is in bold.

**Data set**		**Stubb**	**CSam**	**D2z-set**	**CisModule**	**MCD**
mapping3.adult	34	0.26 (9)	**0.35** (12)	0.26 (9)	0.15 (5)	0.24 (8)
mapping1.adult mesoderm	5	0.20 (1)	**0.60** (3)	0.20 (1)	0.20 (1)	0.20 (1)
mapping1.amnioserosa	7	0.00 (0)	0.29 (2)	**0.43** (3)	0.00 (0)	0.00 (0)
mapping1.blastoderm	77	**0.35** (27)	0.25 (19)	0.27 (21)	0.16 (12)	0.31 (24)
mapping1.cardiac mesoderm	8	0.12 (1)	0.25 (2)	**0.50** (4)	0.12 (1)	0.00 (0)
mapping1.cns	34	0.09 (3)	0.09 (3)	**0.24** (8)	0.09 (3)	0.21 (7)
mapping1.dorsal ectoderm	8	**0.50** (4)	0.12 (1)	0.12 (1)	0.12 (1)	0.25 (2)
mapping1.ectoderm	37	**0.32** (12)	0.30 (11)	0.27 (10)	0.14 (5)	0.16 (6)
mapping2.ectoderm	51	**0.33** (17)	0.31 (16)	0.24 (12)	0.08 (4)	0.20 (10)
mapping1.endoderm	16	**0.19** (3)	**0.19** (3)	**0.19** (3)	0.06 (1)	**0.19** (3)
mapping1.eye	6	**0.33** (2)	**0.33** (2)	0.00 (0)	0.17 (1)	0.17 (1)
mapping2.eye	18	0.22 (4)	0.11 (2)	**0.28** (5)	0.11 (2)	0.17 (3)
mapping1.fat body	5	0.00 (0)	0.00 (0)	0.00 (0)	**0.20** (1)	0.00 (0)
mapping1.female gonad	10	0.10 (1)	0.10 (1)	**0.30** (3)	0.20 (2)	0.20 (2)
mapping1.glia	7	0.14 (1)	**0.43** (3)	**0.43** (3)	0.00 (0)	0.00 (0)
mapping1.imaginal disc	47	0.13 (6)	**0.36** (17)	0.32 (15)	0.11 (5)	0.32 (15)
mapping2.imaginal disc	12	0.00 (0)	0.17 (2)	**0.25** (3)	0.08 (1)	0.17 (2)
mapping3.larva	69	0.16 (11)	0.16 (11)	**0.23** (16)	0.12 (8)	0.10 (7)
mapping1.male gonad	8	**0.25** (2)	**0.25** (2)	0.12 (1)	0.12 (1)	0.12 (1)
mapping1.malpighian tubules	4	**0.25** (1)	0.00 (0)	0.00 (0)	0.00 (0)	0.00 (0)
mapping1.mesectoderm	5	0.20 (1)	**0.40** (2)	0.00 (0)	0.00 (0)	0.00 (0)
mapping1.mesoderm	16	0.38 (6)	0.38 (6)	0.38 (6)	0.12 (2)	**0.44** (7)
mapping2.mesoderm	45	0.27 (12)	0.24 (11)	0.24 (11)	0.11 (5)	**0.29** (13)
mapping1.neuroectoderm	7	**0.43** (3)	0.14 (1)	0.29 (2)	0.14 (1)	0.29 (2)
mapping2.neuronal	54	0.13 (7)	0.20 (11)	**0.26** (14)	0.06 (3)	0.22 (12)
mapping1.pns	24	0.29 (7)	**0.42** (10)	0.21 (5)	0.08 (2)	0.29 (7)
mapping2.reproductive system	20	**0.30** (6)	0.10 (2)	0.25 (5)	0.15 (3)	0.05 (1)
mapping1.salivary gland	6	**0.33** (2)	0.17 (1)	0.17 (1)	0.17 (1)	0.00 (0)
mapping1.somatic muscle	12	0.17 (2)	**0.33** (4)	0.00 (0)	0.08 (1)	0.25 (3)
mapping1.tracheal system	9	0.22 (2)	0.22 (2)	0.11 (1)	0.00 (0)	**0.44** (4)
mapping1.ventral ectoderm	12	**0.25** (3)	0.08 (1)	0.17 (2)	0.17 (2)	0.08 (1)
mapping1.visceral mesoderm	12	0.17 (2)	**0.33** (4)	0.25 (3)	0.00 (0)	0.25 (3)
mapping2.wing	33	0.18 (6)	0.24 (8)	**0.33** (11)	0.09 (3)	0.24 (8)
